# Cerebral hypoxia, missing cortical somatosensory evoked potentials and recovery of consciousness

**DOI:** 10.1186/1471-2377-14-82

**Published:** 2014-04-11

**Authors:** Gustav Pfeiffer, Rüdiger Pfeifer, Stefan Isenmann

**Affiliations:** 1 Abteilung weiterführende Neurorehabilitation, Fachklinik Bad Liebenstein, Kurpromenade 2, 36448 Bad Liebenstein, Germany; 2 Clinic of Internal Medicine I, University Hospital Jena, Erlanger Allee, Germany; 3 Department of Neurology and Clinical Neurophysiology, Helios Hospital Wuppertal, Centre for Clinical Medicine, and University Witten/Herdecke, Wupperrtal, Germany

**Keywords:** Consciousness, Coma, Cardiac arrest, Evoked potentials, Diffusion weighted imaging, Rehabilitation, Minimally conscious state

## Abstract

**Background:**

Bilaterally absent N20 components of the sensory evoked potentials (SEP) from the median nerve are regarded as accurately predicting poor outcome after cardiac arrest.

**Case presentation:**

We are reporting on a patient, who regained consciousness despite this ominous finding. Early after cardiac arrest, MRI showed signal alterations in diffusion weighted imaging (DWI) bilaterally in the primary visual and sensorimotor cortex and in the basal ganglia. SEP were repeatedly absent. The patient survived shut out form sensory and visual experience and locked in for voluntary movements, but kept her verbal competence in several languages.

**Conclusion:**

SEP inform about integrity only of a narrow cortical strip. It is unguarded, but common practice, to conclude from absent SEP, that a patient has suffered diffuse cortical damage after cardiac arrest. Cerebral MRI with DWI helps to avoid this prognostic error and furthers understanding of the sometimes very peculiar state of mind after cardiac arrest.

## Background

It is generally held that patients who have lost cortical somatosensory potentials (SEP) after successful cardio pulmonary resuscitation will never regain consciousness [1,2]. This may become a self fulfilling prophecy if rehabilitative and life sustaining efforts are curtailed. Evidence for this belief is limited. Few patients have been followed for longer than 6 months after arrest [3]. We report on a young adult patient who was comatose without cortical SEP following CPR, yet recovered consciousness later. Coma remission scale (CRS) [4] and interdisciplinary team conferences were prospectively documented every week. DWI was performed 3 days after the event. Information about the patient’s communicative behaviour was provided by a close friend, who studied medicine.

## Case presentation

A promising young architect of 25 years with hereditary long QT syndrome suffered from cardiac arrest during a generalized epileptic seizure in 2003 [5]. Cardiopulmonary resuscitation (CPR) was started immediately. Return of spontaneous circulation was achieved after approx. 30 min. The patient was initially cooled to 33°C for 24 hours and remained comatose after rewarming with a Glasgow Coma Scale of 4 (E1V1M2). Pupils were reactive to light and vestibulo-ocular reflexes were absent. EEG showed no signs of epileptic activity and no burst-suppression pattern. Vigorous generalized myocloni not responsive to valproic acid necessitated continuous deep sedation from days 2 – 14. Neuron specific enolase (NSE) was measured daily for the first five days and reached a maximum at 48.8 ng/mL (n < 12.5 ng/mL) on day 4.

**Figure 1:**
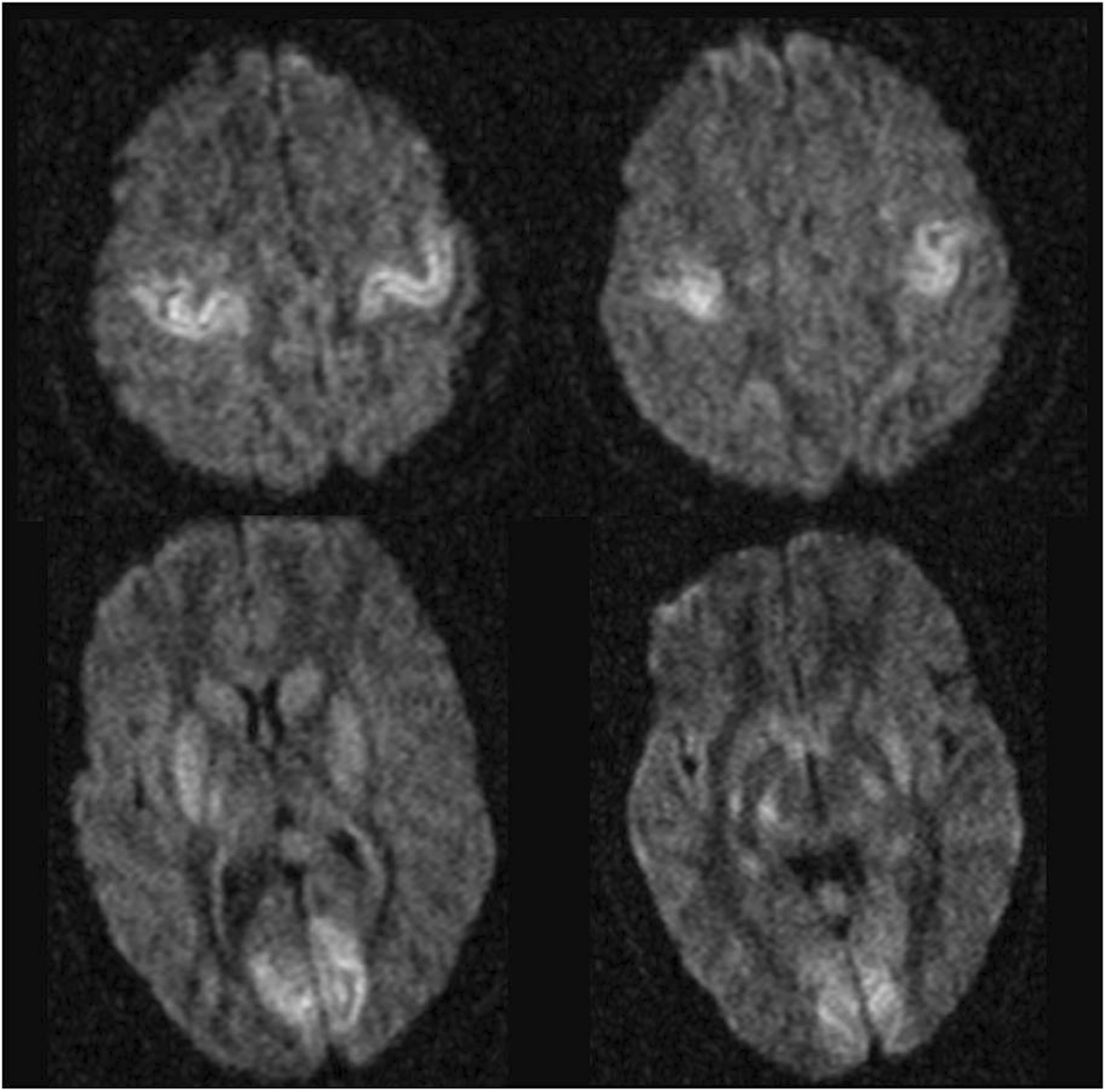
DWI, 3 days after CPR.

**Figure 2:**
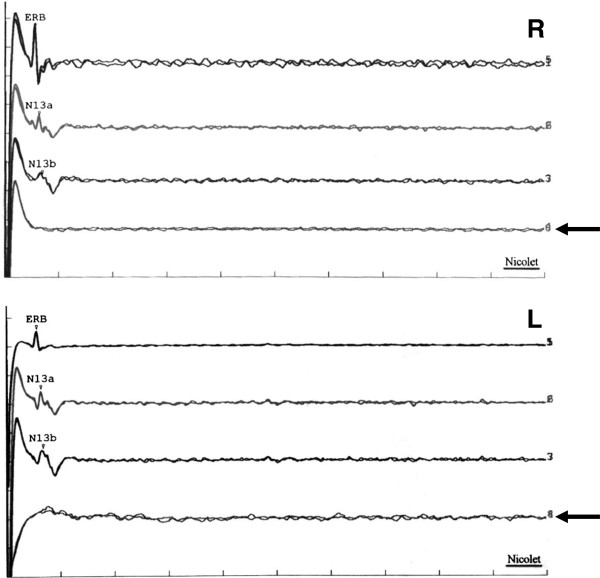
**Median nerve SEP recorded on day 8 after CPR.** Note bilateral absence of cortical N_20_ potentials and late complexes (arrows) while cervical potentials (Erb, N13a, N13b) are preserved (R, right; L, left). Identical results were obtained on day 5.

MRI with DWI two weeks post CPR showed bilateral signal alterations in the primary visual and sensorimotor cortex and in the basal ganglia (Figure 1). N13 was present, but cortical SEP responses after median nerve stimulation were bilaterally absent on days 5 and 8 after CPR (Figure 2). Against the advice of the treating physicians, the family insisted on implantation of a defibrillator to prevent cardiac arrests. At a second attempt of rehabiliation six months after CPR, even gentle touch triggered myoclonic showers and massive flexor spasms which were unresponsive to anticonvulsants and prohibited any therapeutic approach. Continuous transdermal fentanyl, intended as palliative treatment, enabled therapeutic contact and rehabilitation. Seven months after CPR, our patient was able to fixate for several seconds and inconsistently followed moving objects with her eyes. Eight months after CPR, she voluntarily turned her head. Nine months after CPR, she seized food with her lips, answered questions with “a” for “ja [yes]” and “o” for “nein [no]”and welcomed visitors with “ao” for “hallo”. Ten months after CPR, she started to moan persistently. She seemed to realize her condition. Discharge to the family home was planned to facilitate grieving and coping.

Three years after CPR, a friend of her wrote: “there are so many small and large things you should witness: her attention and understanding of situations, her allusions to the past, her jokes, joy, laughing and teasing.” This letter triggered an interview with this friend four years after CPR. At that time our patient had learnt to change her position in bed but still was helpless with her limbs. She was sitting in her wheelchair most of the day. Food had to be put on her tongue. Sometimes she teased her feeders by keeping her mouth closed despite still signalling appetite. After a comment, “you are kidding”, she expressed fun and opened her mouth again. Her signal to finish feeding was mouth closure and facial expression of avulsion. Her repertoire of facial expressions included consternation, surprise, joy, fear, sadness and disgust. When making fun of someone she uttered a staccato laughter “ha, ha, ha”. Except when laughing she was unable to articulate the phoneme “ha”. A very broad “e” accompanied the facial expression of disgust but she was unable to produce an “e” when trying to speak. Consternation was accompanied by humming but she could not hum voluntarily. Talks about previous delightful joint holiday episodes could make her congenially delighted but also sad, suggesting mourning. Thus her responses were not just mirror emotions. She showed dissent when intentionally offered wrong facts such as birthdays or names, followed by joy at correction. Deliberately mispronounced English words provoked fun. She discriminated absurd and meaningful Russian sentences by consternation or assent. She also learned neologisms which elicited joy when used again. A private video shows her happily rocking in her wheelchair while listening to a song. She had favourite songs from before and after her cardiac arrest.

Her mood and attention had deteriorated about half a year before the interview, three and a half years after CPR. She frequently cried heavily for up to twenty minutes, followed by exhaustion. It was impossible to locate the pain by extensive guessing. Eventually a bad tooth was extracted and pain attacks stopped.

Ten years after CPR, one of the authors visited her at home. He was welcomed cheerfully. She confirmed by nodding that she recognized his voice. Her bright mood made it hard to answer in the negative. She had to calm down, before she could negate deliberately wrong letter propositions during a spelling trial of her first name. She happily approved the correct proposal of the first letter. After this success, her joy prevented any further negating which would have included frowning.

Involuntary, mostly horizontal, irregular saccades were present almost continuously. They became chaotic with agitation. Questions about her visual experience provoked only vague responses. Her brother had noticed that she lost attention for television when he turned off the volume.

## Conclusion

It is easy to overlook cognitive capacities in unresponsive wakeful patients [6]. Five of 54 patients in a vegetative or minimal conscious state were able to comply with motor and spatial imaging tasks during functional MRI [7]. Two of these five patients are described in detail. The seminal patient of this series [8] showed behaviours ranking not higher than 13 of 62 according to the Wessex Head Injury Matrix [9] at the time of functional MRI. This level is in the range of basic early behaviours of coma-recovery, which are thought to be mostly reactive [9]. The patient had a unilaterally preserved N_20_ component of the SEP. The second detailed description (7, suppl. inf.) deals with a patient who was considered to be in a vegetative state after specialized thorough assessment for one month. At the time of the functional MRI eighteen months later, only 6 of 10 standardized assessments yielded inconsistent responses to some commands, but no functional or intentional communication. The risk of overlooking cognitive capacities was even higher in our patient because generalized myoclonus precluded any assessment. This myoclonus was neither classifiable as generalized early myoclonus which is a transient phenomenon occurring within one day after resuscitation, nor as chronic hypoxic myoclonus which is an action myoclonus associated with voluntary movements [10]. It was an unusual phenomenon, but together with the missing cortical sensory evoked potentials it was thought to be inauspicious. Moreover, recovery of consciousness was deemed extremely unlikely later than three months after non-traumatic brain injury, before the availability of functional MRI [11].

Our patient survived only because her parents insisted on continued intensive care treatment despite the firm conviction of her neurologists that she would remain unconscious because of absent cortical SEP responses. This “accurate” predictor of poor outcome has an estimated false positive ratio of 0.7% (95% confidence interval 0.1 to 3.7) [3], even after therapeutic hypothermia [12]. The MRI shows why the SEP had been misleading in our patient: the primary sensory cortex, the generator of the N_20_-component of the SEP, was one of the few cortical areas with altered DWI signals. It is clearly evident, but frequently neglected, that the N_20_ component of the SEP informs only about a narrow cortical strip. To conclude from its absence, that there is diffuse cortical damage is an unguarded but common practice after cardiac arrest.

Recently, a similarly astonishing regain of consciousness has been reported in a patient after CPR who had no N_20_ SEP responses bilaterally [13] and recovered functionally, whereas our patient remained dependent, but recovered consciousness. This patient was part of a retrospective series of 113 consecutive patients entering a German specialised rehabilitation unit comatose, vegetative or minimally conscious about one month after CPR [14]. Five of these patients with bilaterally absent N_20_ (“malignant SEP” in Table two in [14]) recovered consciousness to CRS scores above 23, which means that they used objects purposefully, recognized familiar people and spoke at least simple words (Table two in [14]). The prognostic indicators for early prognosis of CPR may be not applicable after prolonged survival.

SEP findings should also be interpreted with more caution because they seem to be less reliable than expected. Five neurologists agreed only moderately about presence of the N_20_ peak after cardiac arrest [15]. In a recent study [16] the expert agreement was “very good” (kappa-coefficient: 0.88) only for well preserved SEP patterns. For patterns predicting bad outcome, kappa was only 0.76 (“good”). With a specificity of 93.5% the pattern of bilaterally absent N_20_ predicted poor outcome less accurately than previously estimated [3]. This unexpected suboptimal reliability warrants prognostic modesty after cardiac arrest.

Prognostic assessment after cardiac arrest should include DWI. An extensive cortical MRI lesion pattern was associated with poor outcome in 22 consecutive patients, whereas a regional pattern as in our patient was less ominous [17]. Less than 10% of the brain volume had reduced absolute diffusion coefficients in survivors of post-cardiac arrest, whereas 10 to 40% of the brain was involved in most patients who died Figure four in ref. [18]. There are reports of symmetrical regional MRI lesion patterns, similar to our patient [17-19], Figure three in ref. Another patient with early myoclonic status epilepticus after CPR and therapeutic hypothermia who recovered despite several additional unfavourable prognostic signs had clinical abnormalities disproportionately worse than her DWI abnormalities-which were regional and symmetrical like in our patient, but did not include the primary sensory cortex [20]. She had preserved cortical SEP responses.

Our patient’s MRI helps to understand her peculiar cognitive situation: locked in for voluntary movements and locked out form a great deal of sensory experience. Our patient’s auditory cortex was the only primary sensory cortex with unaltered diffusion on MRI, explaining why sophisticated auditory games were her principal intellectual pleasure. We have few clues to our patient’s visual experience but we can conjecture about her somatosensory experience. She suffered badly from a toothache, but was unable to locate it. The sensory-discriminative components of pain are mediated by the primary and secondary sensory cortex, whereas the anterior cingulum is involved in affective evaluation of noxious stimuli [21]. Dissociation of the two pain components has been observed in a communicating patient [22]. It is probably present in our patient who has lost her primary sensory cortex, but preserved her anterior cingular cortex. There is a similar dissociation in her expressive behaviour. As in the anterior operculum syndrome [23] she is unable to articulate phonemes voluntarily, but utters them as part of expressed emotions.

Our patient recovered consciousness to a considerable degree, however its content [24] is limited: her toothache obviously as not part of her discriminative consciousness. She probably suffers from her speechlessness more than from her quadriplegia because she is conscious of her failing acoustic output, but perhaps unaware of her limbs. The fate of our patient shows that MRI early after cardiac arrest is not only a valuable prognostic tool, but also helps to understand the cognitive state of patients after cerebral hypoxia.

### Consent

Written informed consent for publication of this Case report and accompanying images was obtained from the patient’s mother as her guardian. A copy of the written consent is available for review by the Editor of this journal.

## Competing interests

The authors declare that they have no competing of interest.

## Authors’ contributions

SI and RP were responsible for the acute care of the patient, and GP for rehabilitation and follow up. GP prepared the manuscript. It was critically revised by SI and RP. All authors read and approved the final manuscript.

## Pre-publication history

The pre-publication history for this paper can be accessed here:

http://www.biomedcentral.com/1471-2377/14/82/prepub
